# Circadian Features of Neutrophil Biology

**DOI:** 10.3389/fimmu.2020.00576

**Published:** 2020-04-03

**Authors:** Alejandra Aroca-Crevillén, José M. Adrover, Andrés Hidalgo

**Affiliations:** ^1^Department of Cell and Developmental Biology, Centro Nacional de Investigaciones Cardiovasculares, Carlos III, Madrid, Spain; ^2^Institute for Cardiovascular Prevention (IPEK), Ludwig Maximilians University, Munich, Germany

**Keywords:** neutrophil, circadian inflammation, chronotherapy, molecular clock, oscillatory signals

## Abstract

Rhythms in immunity manifest in multiple ways, but perhaps most prominently by the recurrent onset of inflammation at specific times of day. These patterns are of importance to understand human disease and are caused, in many instances, by the action of neutrophils, a myeloid leukocyte with striking circadian features. The neutrophil's short life, marked diurnal variations in number, and changes in phenotype while in the circulation, help explain the temporal features of inflammatory disease but also uncover core features of neutrophil physiology. Here, we summarize well-established concepts and introduce recent discoveries in the biology of these cells as they relate to circadian rhythms. We highlight that although the circadian features of neutrophils are better known and relevant to understand disease, they may also influence important aspects of organ function even in the steady-state. Finally, we discuss the possibility of targeting these temporal features of neutrophils for therapeutic benefit.

## General Features of Circadian Immunity

The rotational period of the Earth creates variations in sunlight exposure and provides regular diurnal cues to organisms [1]. Sensing of these cues synchronizes organismal physiology and behavior with external conditions [2], thus providing an evolutionary advantage by allowing organisms to anticipate and adapt to a changing environment [1]. These cyclic biological changes create circadian rhythms which are endogenous, self-maintained oscillations that display a periodicity of ~24 h [3]. These rhythms are entrainable by periodic changes in environmental cues, such as light or food [4, 5]. Central and peripheral mechanisms regulating circadian oscillations in organisms and in cells have been reviewed extensively, including in the immune system, and will not be further reviewed here [6, 7].

Circadian rhythms are present in many cellular and humoral components of the immune system. Granulocytes and monocytes exhibit circadian oscillations in their numbers in blood, both in humans [8] and mice [9], and these oscillations are also robust in T- and B-lymphocytes [10–12]. Circadian variations in clock gene expression have also been reported in many types of immune cells, including monocytes [13, 14], macrophages [15, 16], neutrophils [17, 18], dendritic cells [12], or lymphocytes [10, 12]. This suggested the presence of functional, intrinsic clockworks in immune cells, and recent studies have demonstrated that many immune processes are under direct circadian control. For example, rhythmic leukocyte recruitment is regulated by circadian expression of pro-migratory factors within endothelial cells [9], circadian trafficking of lymphocytes through lymph nodes is controlled by Bmal1-dependent expression of the receptor CCR7 [19], and the response of phagocytes to *Leishmania* infection is abolished in mice lacking the molecular clock in innate immune cells [20]. Overall, these and many other observations have expanded the ascribed role of circadian clockworks and oscillatory signals within the microenvironment in the control of immune cell trafficking and host-pathogen interactions. Consistent with variations in the immune cell number and function, inflammatory diseases display circadian manifestations. Prominent among these are those that affect the cardiovascular system, with acute vascular events displaying rhythmic patterns in both onset and severity. For instance, myocardial infarction in mice and humans shows circadian variations in both onset (for humans) and infarct size depending on the time of day, with evidence supporting changes in leukocyte infiltration rates into the myocardium [17, 21–23]. Occurrence of ischemic stroke also has a peak of incidence in the morning [24], in coincidence with higher atherosclerotic plaque rupture at this time [25]. Likewise, certain autoimmune disorders such as rheumatoid arthritis exhibit daily variations in joint inflammation with stiffness and pro-inflammatory cytokines peaking in the morning [26, 27]. Finally, sepsis modeled by caecal ligation or lipopolysaccharide (LPS) injection also shows circadian variations, with increased severity during the night in mice [9, 28]. Below, we focus our discussion on the circadian properties of neutrophils, a type of leukocyte whose short life cycle appears to have adapted optimally and in multiple ways to the circadian rhythms of mammals [29, 30].

## Circadian Features of Neutrophils in the Bone Marrow

**Figure 1:**
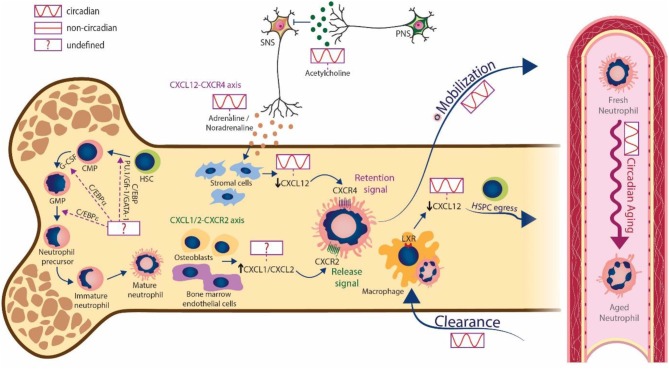
Circadian regulation of neutrophils in bone marrow and blood. Mature neutrophils are produced in the bone marrow during granulopoiesis. The transcription factors PU.1, Gfi-1, GATA-1 and different enhancer-binding proteins (C/EBPs) are involved in this process, but the existence of oscillatory changes in their expression is unknown. The sympathetic nervous system releases cues (adrenaline and noradrenaline) that act on stromal cells to generate circadian changes in CXCL12 levels. This ultimately decreases the expression of CXCL12 and promotes the circadian release of neutrophils into the bloodstream. In turn, the parasympathetic nervous system suppresses the activity of the SNS trough cholinergic signals (acetylcholine). In addition to the CXCR4/CXCL12 axis, signaling through CXCR2 by the chemokines CXCL1 and CXCL2 produced by osteoblasts and bone marrow endothelial cells, also mediates neutrophil egress, however the circadian regulation of this axis needs further investigation. Neutrophils undergo aging in circulation following circadian patterns and are finally cleared into the bone marrow and other tissues. The engulfment of aged neutrophils by macrophages activates LXR signaling, which in turn blunts expression of CXCL12 to promote the circadian egress of hematopoietic stem and progenitor cells (HSPCs). Note that cell morphologies are characteristic from mice.

Neutrophils are mainly produced within the bone marrow (BM) through a process known as *granulopoiesis*. A complex interplay between the transcription factors PU.1, enhancer-binding proteins (C/EBPs), Gfi-1 and GATA-1 determines the commitment of immature progenitors to the myeloid-lineage [reviewed in [31]]. From this point on, C/EBPα induces the expression of the granulocyte colony stimulating factor receptor (G-CSFR/CSF3R), which allows signals delivered by the cytokine granulocyte-colony stimulating factor (G-CSF/CSF3) to promote granulopoiesis [32]. Insights based on single cell analyses have additionally defined committed neutrophil precursors reliant on the transcription factor C/EBPε [33, 34] (Figure 1). Recent reviews have already described basic aspects of granulopoiesis, including the different stages of maturation both under homeostasis or emergency [30, 35, 36], and will not be discussed here further. A more detailed characterization of the signals that control these developmental stages will be needed to determine the possible existence of a circadian component that boosts granulopoiesis at certain times of the day.

The BM maintains a neutrophil pool ready to be released under homeostatic and stress conditions. Given the toxic potential of neutrophils, granulopoiesis, and subsequent release must be tightly regulated to balance their numbers in the circulation. This is achieved by massive daily production [up to 2 × 10^11^ cells per day in humans [37]] and temporally-gated release into blood [38]. Circulating neutrophils ultimately infiltrate tissues after only 6–10 h in the circulation, which makes them one of the shortest-lived cells in our bodies. It is noteworthy that the percentage of mature neutrophils in human and mouse peripheral blood is between 50–70 and 10–25%, respectively [30]. These differences can make difficult to accurately translate findings across species. Under homeostatic conditions immature neutrophils are largely absent from the circulation in both human and mice [33]. However, under inflammatory conditions the number of immature neutrophils increases in blood, as shown by the increase of the CD101-negative population in the circulation of tumor-bearing mice [33] or the release of CD16-dim neutrophils from the BM in a model of human endotoxemia [39].

Oscillatory signals within the BM are believed to play an important role in both release and clearance. Studies in mice have shown that the chemokine CXCL12, acting through its receptor CXCR4, provides a key retention signal for neutrophils (and other cells) within the BM [40, 41]. Importantly, regulation of CXCL12 levels in the mouse BM appears to be controlled by neural signals. Sympathetic nerves that innervate the BM deliver diurnal adrenergic signals to stromal cells through β3-adrenergic receptors, which inhibit CXCL12 expression and generate oscillatory expression of the chemokine [42]. Cholinergic signals from the parasympathetic nervous system (PNS), in turn, have been shown to inhibit adrenergic activity of the murine SNS at night [43], altogether establishing tight temporal patterns in the BM. In mice, downregulation of CXCL12 at daytime drives the circadian egress of HSCs [42], and the release of neutrophils at this same time also coincides with decreased CXCL12 [40] (Figure 1). Several lines of evidence suggest that timed release through CXCL12 underlies the circadian variations of neutrophil numbers in blood: first, administration of a CXCR4 antagonist mobilizes neutrophils from the BM in both mice [44] and humans [45, 46], although another study found that the antagonist mobilized neutrophils from the lungs of both mice and macaques [47]; second, genetic deletion of *Cxcr*4 only in myeloid cells results in massive neutrophilia in blood [41], and interestingly also results in blunted oscillations of neutrophils in blood [17]. In addition to the CXCL12/CXCR4 axis, CXCR2 also plays an important role in neutrophil trafficking [44, 48] as its absence leads to neutrophil retention in the BM, and partly counteracts CXCR4 to regulate the egress of neutrophils [49]. Interestingly, the CXCR2 ligands CXCL1 and CXCL2 are constitutively expressed by BM endothelial cells and osteoblasts [49] (Figure 1), and their expression can be also enhanced by external stimuli, including G-CSF [49] or thrombopoietin [50], thus contributing to neutrophil mobilization. Together, these results highlight the tight regulation of cues driving neutrophil egress, all of which are likely subjected to circadian control in a manner similar to CXCL12, but this needs to be explored further.

After circulating for several hours, mature neutrophils are ultimately cleared in different tissues (see discussion below). Among these, it is interesting that the BM is one of the major clearance sites for neutrophils [51, 52] in a process also controlled by the CXCL12/CXCR4 pathway in both humans and mice [41, 44, 48, 53]. Adoptive transfer experiments showed that the population of neutrophils that preferentially homes to the BM expresses higher levels of CXCR4, which agrees with the notion that CXCR4^HI^ aged neutrophils (those that have remained longer in the circulation; see below) gain tropism for the BM as part of their programmed lifecycle [44]. There are, however, contradictory observations as homing experiments indicated that CXCR4^HI^ aged neutrophils have a similar ability to infiltrate the BM as CXCR4^LO^ neutrophils [53]. In addition to neutrophil-intrinsic changes, other studies have shown the importance of environmental factors in modulating the circadian recruitment of leukocytes into tissues under homeostatic conditions. For example, expression of P- and E-selectins as well as VCAM-1, controlled by the SNS, oscillate in a circadian fashion in the BM vasculature, and favor the recruitment of leukocytes at night in mice [9].

Adding to these circadian aspects of neutrophil trafficking to and from the BM, clearance of neutrophils in this organ has been shown to regulate the hematopoietic niche. Studies in mice showed that aged neutrophils entering the BM are engulfed by BM macrophages [51]. Through a process dependent on the LXR nuclear receptors, these cells trigger reductions in CXCL12 expression and alter the cellular composition of the hematopoietic niche, altogether promoting the egress of hematopoietic stem cells (HSCs) into the circulation [53] (Figure 1). The return of neutrophils to the BM at the end of the resting phase (in mice; between ZT5 and ZT13) is promoted by SNS-dependent, circadian regulation of CXCL12 and other molecules required for HSC homing [9]. These highly-regulated processes contribute to control neutrophil numbers and the properties of the BM throughout the day.

## Clock-Driven Physiology of the Circulating Neutrophil

The remarkably short lifespan of neutrophils in blood implies that many resources must be employed in their production [54]. This feature likely relates to evolutionary trade-offs for a cell that is key to immune defense, but is also highly cytotoxic and can incite vascular inflammation [55]. A consequence of this rapid turnover is that neutrophil numbers follow strong circadian changes in blood. Remarkably, these circadian oscillations also affect the phenotype of circulating neutrophils, a property referred to as neutrophil *aging* [29]. Most data on neutrophil aging classically derived from *in vitro* studies revealing, for example, increased surface levels of CXCR4 [44], and decrease of CXCR2 [49] or L-selectin [56]. The physiological impact of this circadianally-regulated phenomenon, and the underlying molecular mechanisms, have remained unclear until recently.

Using a model of neutrophil transfer into antibiotic-treated mice, a study proposed that neutrophil aging is controlled by extrinsic, microbiota-derived and TLR4-dependent signals [57]. A caveat of these studies was that no links were established with actual circadian timing, thus making the temporal relevance of the findings unclear. More recently, another study reported that neutrophil aging was controlled cell-intrinsically by the core clock gene *Arntl* (encoding Bmal1), was entrained by light, and was dependent on antagonistic CXCR2 and CXCR4 signaling [17]. Interestingly, neutrophil-intrinsic Bmal1 regulated the circadian expression of CXCL2, a chemokine that signaled in an autocrine fashion through CXCR2 to promote the transcriptional and phenotypic changes associated with neutrophil aging. In the proposed model, CXCR4 counteracted signaling through CXCR2, thereby blocking this program [17]. This is consistent with studies in humans showing circadian variations in plasma levels of CXCL12 (the ligand for CXCR4) in antiphase with the aging phenotype [17, 18], although it is noteworthy that these studies found that the levels of BMAL1 are very low in human peripheral neutrophils. Thus, circadian neutrophil aging appears to be cell-intrinsically regulated by the molecular clock, and extrinsically through CXCR4 signaling. Because the same chemokine receptors that control the egress of neutrophils from the BM [49] also control aging, we propose that CXCR4 signaling temporally coordinates the release of neutrophils into blood with the onset of aging only in peripheral blood, possibly protecting the BM from the potentially toxic activity of activated neutrophils. Since CXCR4/CXCL12 signaling is controlled circadianally by sympathetic signaling under steady-state conditions [9, 58] it will be interesting to explore how chronic or acute inflammation alter the circadian properties of neutrophils.

An intriguing finding from these studies was that aged neutrophils are preferentially cleared out from the circulation into healthy tissues under steady-state conditions [17], whereas non-aged cells (“fresh” neutrophils) are preferentially recruited to inflammatory sites. This was explained by the progressive loss of microvilli needed for efficient rolling as neutrophils aged over time [17]. Further, expression of CXCR2 is reduced in aged neutrophils and we have recently shown that these cells feature reduced granule content and NET-forming capacity relative to fresh neutrophils [59], altogether suggesting blunted inflammatory properties for aged neutrophils. However, these blunted inflammatory properties are in apparent contradiction with reports showing elevated inflammogenic properties of aged neutrophils in mice [57], as well as increased adhesion, ROS production and phagocytic capacity in human aged neutrophils [18]. The reason for these discrepancies deserves further investigation.

The pathophysiological consequence of this clock-controlled behavior of circulating neutrophils has been put in manifest in the context of infection and vascular inflammation. For example, enhanced seeding of tissues like the kidney by (aged) neutrophils at night protected from fungal infection. In a model of *Candida albicans* infection, pathogen clearance was superior at night, a time when neutrophils had already entered the tissue, and exaggerated neutrophil aging by deletion of CXCR4 markedly protected against infection. In contrast, deletion of *Arntl* rendered mice more susceptible to infection at night [17]. Similar outcomes were found in the context of sterile inflammation, as the circadian differences in ischemic stroke or myocardial infarction were also sensitive to the deletion of Bmal1 in neutrophils [17]. An interesting conclusion from these experiments is that neutrophil numbers in blood, which have been correlated with vascular disease and used for prognosis in the clinics [60], may not be the key factor in disease outcome while, at least in mice, the aging phenotype of the cells is a better predictor of the immune response.

## Neutrophil in Tissues and Circadian Pathophysiology

**Figure 2:**
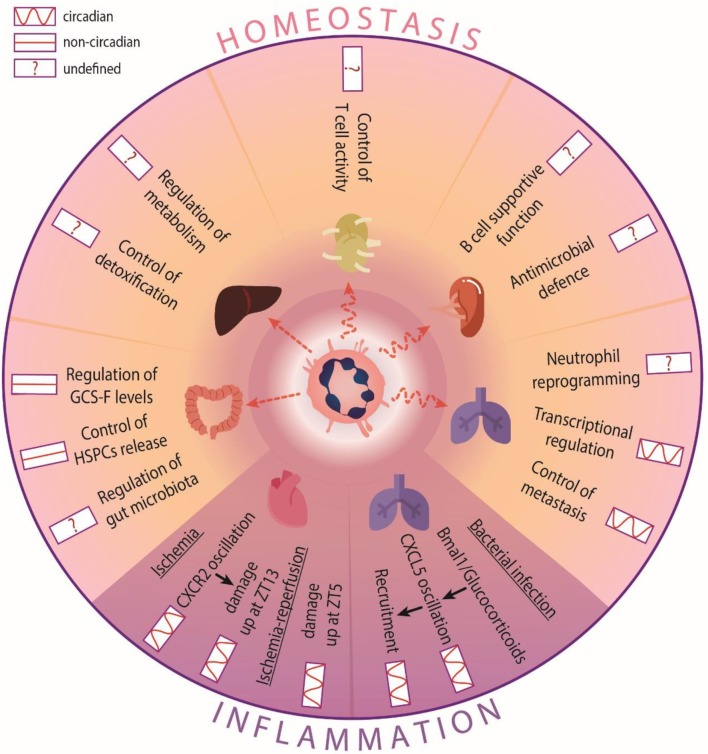
Circadian functions of neutrophils in tissues. In homeostasis, neutrophil infiltration into most tissues is circadian, but not in the intestine or the liver. Neutrophils perform different functions in a circadian-independent manner: In the intestine, they control G-CSF production to mobilize HSC. In other tissues they regulate circadian processes, such as transcriptional programs and tumor invasion in the lung. The circadian influence in other tissues is not well-defined. For example, metabolism or detoxification control in the liver, gut microbiota regulation in the intestine, or their own re-education and immune defense in the spleen or lymph nodes, respectively. In inflammatory scenarios, neutrophil recruitment oscillates and influences disease outcome. During bacterial infection in the lung, bronchiolar cells modulate CXCL5 expression to control the oscillatory recruitment of neutrophils. In models of cardiac ischemia, increased infiltration into the heart accounts for exacerbated cardiac damage at different times depending on the type of injury performed.

Commonly believed to be eliminated only in the BM, spleen and liver [61], neutrophils have now been shown to infiltrate many other tissues in the steady-state (at least in mice), including the intestine, lung, white-adipose tissue (WAT), skin, skeletal muscle, lymph nodes, kidneys and heart [52]. Notably, infiltration of neutrophils into most naïve tissues follows circadian patterns with a peak at night, with exceptions in the intestine, liver and WAT in which no circadian oscillations were detected [52] (Figure 2). Remarkably, the function of neutrophils in most of these tissues remains virtually unexplored.

An outstanding question is whether tissue-infiltrating neutrophils organize in specific areas that enables particular functions in each tissue. In the intestine, for example, neutrophils distribute in clusters around isolated lymphoid follicles, and are surrounded by CD169+ macrophages [52]. In this case, the proximity to IL-23-producing cells predicted regulation of the levels of this cytokine and downstream production of G-CSF, an important mobilizing cytokine. Indeed, we identified a role for gut-infiltrating neutrophils in regulating systemic G-CSF levels and subsequent mobilization of hematopoietic stem and progenitor cells (HSPCs) from the BM [52] (Figure 2). Intriguingly, this regulatory role appeared to be unrelated of the circadian release of HSPC in blood [42, 53]. Whether neutrophils in the gut coordinate with other oscillatory signals within this environment, such as the intestinal microbiota [62], remains to be explored.

Contrary to the gut, neutrophil infiltration of the lungs follows tight circadian patterns [52]. The need to defend against colonizing bacteria may explain the existence of a large marginated pool of neutrophils within the lung microcirculation, as shown both in human and mice [63]. In mice, the lung has been proposed to be an “education” site for neutrophils incoming from injured tissues to promote their return to the BM [64]. Interestingly, in the mouse lungs neutrophils were shown to entrain global circadian transcription that appeared to predispose to organ invasion by metastatic cells [52], thereby suggesting that diurnal neutrophil clearance in the lung may influence the temporal dynamics of patho-physiological processes. The mechanisms underlying diurnal regulation of circadian expression in this tissue, and whether this could be extended to other tissues, remains unknown. Reciprocal regulation is also possible, and indeed extrinsic regulation of circadian recruitment of neutrophils during bacterial infection is potently mediated by bronchiolar cells, whose expression of attractant chemokine CXCL5 is regulated by glucocorticoids and Bmal1 in a circadian manner [65] (Figure 2).

The liver is a preferential site for neutrophil elimination [30] (Figure 2). In this organ, many physiological functions such as energy metabolism or detoxification are under circadian control [66] and disruption of the hepatic clock promotes disease, including cirrhosis, hepatic steatosis and liver cancer [66], some of which have been shown to be regulated by neutrophils [67]. Whether timed infiltration of neutrophils in this tissue influences these or other physiological liver functions remains to be explored.

In hematopoietic and lymphoid tissues other than the BM, such as the spleen, neutrophils have been reported to promote maturation, differentiation, and antibody production of B cells [68]. Neutrophils at different stages of maturation have also been postulated to perform antimicrobial functions in the spleen, and facilitate clearance of *Streptococcus pneumonia* [69]. Recently, a study has shown that MHCII+ neutrophils in lymph nodes interact with dendritic cells and macrophages, presumably to modulate T cell activation (Figure 2), with the caveat that this was largely shown *ex vivo* using bone marrow-derived cells [70]. Given the rhythmic recruitment of neutrophils to both spleen and lymph nodes [52, 71], it is conceivable that certain aspects of adaptive immunity and antimicrobial properties of these organs are circadianally regulated by neutrophils, but this too needs further investigation.

A key question is what dictates the temporal pattern of neutrophil entry into the different tissues. A recent study found that cell-intrinsic signals regulated by Bmal1, as well as environmental oscillations of migratory factors orchestrate the rhythmic trafficking of neutrophils and other leukocytes to different tissues in both homeostatic and inflammatory conditions, in human and mice [71]. Blockade experiments performed at ZT13, coinciding with neutrophil exit from the circulation, showed that CXCR4 and ICAM-1 in the BM, L-selectin in the lymph nodes and spleen, and VCAM-1 in the liver, are the main factors that guide neutrophil emigration to those tissues, with oscillations in both neutrophils or endothelial cells [71]. These detailed studies raise the possibility of exploiting this circadian signature of migration for chronotherapy.

The rhythmic recruitment of neutrophils could be also responsible for the circadian manifestation of various inflammatory diseases. In a model of myocardial ischemia, the exacerbated infiltration of neutrophils at night (ZT13) accounted for increased cardiac damage at this time [22]. In this case, the differential recruitment appeared to be CXCR2-dependent [22]. This correlates with data showing increased CXCR2 expression on neutrophils at night [17]. Interestingly, in the context of myocardial ischemia-reperfusion, infarct sizes were larger in the morning [17, 23], suggesting that the type of injury (ischemic or after reperfusion) follows distinct circadian patterns. Altogether, these studies uncovered the importance of circadian neutrophil infiltration across different tissues, with potential implications in the treatment of inflammatory disease (Figure 2).

## Targeting the Circadian Properties of Neutrophils for Therapy

Several studies have demonstrated the importance of the (circadian) time parameter in clinical settings, which raised the possibility of using these temporal physiological features for therapeutic benefit (i.e., chronotherapy). Indeed, administration of drugs at specific times of day in diseases such as cancer or asthma, or the performance of surgical procedures at specific times, has often resulted in enhanced therapeutic success [7]. These findings highlight the possibility of “personalizing” medicine at the temporal level.

The historical reticence to target neutrophils therapeutically is explained by their essential antimicrobial function. However, a wealth of studies in the past few years have identified heterogeneity among neutrophils, raising the possibility of targeting only specific, disease-causing subsets. In arthritic mice, G-CSF receptor blockade decreases disease progression by inhibiting neutrophil accumulation and local production of pro-inflammatory cytokines without affecting their defensive function [72]. *In vivo* interference with the production of neutrophil extracellular traps (NETs) has been shown to be protective in systemic lupus erythematosus (SLE) [73] and transfusion-related acute lung injury (TRALI) [74], whereas the β1-adrenergic-receptor antagonist metoprolol decreases infarct size during AMI by interfering neutrophil recruitment and neutrophil-platelet interactions [75]. These and other examples postulate the possibility to target neutrophils therapeutically, as reviewed recently [76, 77]. Given the observation that the molecular clock influences the effector functions of neutrophils, an outstanding question is whether this “neutrophil clock” can be targeted to prevent inflammatory disease.

An extensive transcriptomic study focused on rhythmic gene expression in whole tissues revealed that many common anti-inflammatory drugs, which in turn have short half-lives, can be directed to circadian genes or their products, thus pointing out the potential therapeutic benefit of targeting clock genes and dosing clock-directed drugs at optimal times to improve their effectiveness [78]. As an example, disruption of the circadian clockwork in macrophages eliminates the exacerbated endotoxin-induced cytokine response observed at night by suppressing the expression of the circadian repressor REV-ERBα [79]. This is of importance since a synthetic REV-ERB ligand (GSK4112) was shown to attenuate cytokine production by macrophages [79], in what was one of the first proof of concept studies that targeted molecular clock proteins to modulate inflammation. This aligned with studies showing the beneficial effect of the REV-ERB agonist SR9009 in reducing atherosclerotic plaque size in LDL receptor-deficient mice [80]. In addition, *in vitro* targeting of the repressor clock protein CRY with the activator KL001 also demonstrated anti-inflammatory effects in chronic arthritis [27]. Despite the *in vitro* nature of these reports, clock-mediated therapy for immune-mediated diseases emerges as a valuable therapeutic tool, which we expect will be soon also exploited in neutrophils.

Although the full extent of the physiological consequences of circadian rhythms in neutrophils is still unclear, recent studies have suggested its therapeutic potential. For example, disruption of Bmal1 in club cells and adrenalectomy in the context of circadian recruitment of neutrophils to lungs after LPS revealed blunted glucocorticoid signaling, non-rhythmic expression of CXCL5, neutrophilia and antimicrobial responses [65]. Other studies tested the possibility of targeting the circadian properties of neutrophils and monocytes in atherosclerosis, by showing arterial- and time-specific repression of leukocyte recruitment to plaques upon inhibition of CCR2 [81]. Timed CCR2 blocking at nighttime (ZT17) decreased arterial myeloid cell recruitment and atherosclerotic lesion formation, whereas the neutralization at daytime (ZT5) had no effect, suggesting again the importance of chrono-pharmacology-based approaches [81]. Finally, targeting pro-migratory factors VCAM-1, ICAM-1 and CD49d during inflammatory challenge with LPS affected neutrophil trafficking and blunted inflammation [71]. Overall, these findings have provided strong evidence that targeting circadian mechanisms specific to the immune system may have therapeutic value. Moving forward, we propose that complete characterization of the circadian features of neutrophils, including our recent identification of a cell-intrinsic circadian “timer” [17], will yield powerful new strategies to bring time, and immunity, on the patient's side.

## Author Contributions

All authors contributed equally to the writing of this review. AA-C prepared figures. JA wrote a section of the review. AH coordinated the writing and edited the text and figures.

### Conflict of Interest

The authors declare that the research was conducted in the absence of any commercial or financial relationships that could be construed as a potential conflict of interest.
